# High childhood serum triglyceride concentrations associate with hepatocellular adenoma development in patients with glycogen storage disease type Ia

**DOI:** 10.1016/j.jhepr.2022.100512

**Published:** 2022-05-29

**Authors:** Martijn P.D. Haring, Fabian Peeks, Maaike H. Oosterveer, Martijn C.G.J. Brouwers, Carla E.M. Hollak, Mirian C.H. Janssen, Janneke G. Langendonk, Alexander J.M. Rennings, Margreet A.E.M. Wagenmakers, Henkjan J. Verkade, Terry G.J. Derks, Vincent E. de Meijer

**Affiliations:** 1 Department of Surgery, University of Groningen, University Medical Center Groningen, Groningen, the Netherlands; 2 Department of Metabolic Diseases, Beatrix Children’s Hospital, University of Groningen, University Medical Center Groningen, Groningen, the Netherlands; 3 Department of Pediatrics, Center for Liver Digestive and Metabolic Diseases, University of Groningen, University Medical Center Groningen, Groningen, the Netherlands; 4 Department of Internal Medicine, Division of Endocrinology and Metabolic Disease, Maastricht University Medical Center+, Maastricht, the Netherlands; 5 Department of Endocrinology and Metabolism, Amsterdam University Medical Center, University of Amsterdam, Amsterdam, the Netherlands; 6 Department of Internal Medicine, Radboud University Medical Centre, Nijmegen, the Netherlands; 7 Department of Internal Medicine, Center for Lysosomal and Metabolic Diseases, Erasmus University Medical Center, University Medical Center Rotterdam, Rotterdam, the Netherlands; 8 Pediatric Gastroenterology and Hepatology, Department of Pediatrics, Beatrix Children’s Hospital, University of Groningen, University Medical Center Groningen, Groningen, the Netherlands

**Keywords:** Benign neoplasm, Hepatic adenoma, Glycogen storage disease type Ia, Metabolic disorder, Glucose-6-phosphatase triglycerides, GSDIa, glycogen storage disease type Ia, *G6PC1*, glucose-6-phosphatase catalytic subunit 1, HCA, hepatocellular adenoma, G6Pase, glucose-6-phosphatase, TG, serum triglyceride concentration, MRI, magnetic resonance imaging, PSV, predicted severe variant, HR, hazard ratio

## Abstract

**Background & Aims:**

Glycogen storage disease type Ia (GSDIa) is an inborn error of carbohydrate metabolism caused by pathogenic variants in the glucose-6-phosphatase catalytic subunit 1 (*G6PC1*) gene and is associated with hepatocellular adenoma (HCA) formation. Data on risk factors for HCA occurrence in GSDIa are scarce. We investigated HCA development in relation to sex, *G6PC1* genotype, and serum triglyceride concentration (TG).

**Methods:**

An observational study of patients with genetically confirmed GSDIa ≥12 years was performed. Patients were categorised for sex; presence of 2, 1, or 0 predicted severe *G6PC1* variant (PSV); and median TG during childhood (<12 years; stratified for above/below 5.65 mmol/L, *i.e.* 500 mg/dl).

**Results:**

Fifty-three patients (23 females) were included, of which 26 patients developed HCA at a median (IQR) age of 21 (17–25) years. At the age of 25 years, 48% of females and 30% of males had developed HCA (log-rank *p* = 0.045). Two-thirds of patients with GSDIa carried 2 PSVs, 20% carried 1, and 13% carried none. Neither the number of PSVs nor any specific *G6PC1* variants were associated with HCA occurrence. Childhood TG was 3.4 (3.0–4.2) mmol/L in males *vs*. 5.6 (4.0–7.9) mmol/L in females (*p* = 0.026). Childhood TG >5.65 mmol/L was associated with HCA development at younger age, compared with patients with childhood TG <5.65 mmol/L (18 *vs*. 33 years; log-rank *p* = 0.001). Cox regression analysis including TG, sex, and TG–sex interaction correction revealed childhood TG >5.65 mmol/L as an independent risk factor for HCA development (hazard ratio [HR] 6.0; 95% CI 1.2–29.8; *p* = 0.028).

**Conclusions:**

In patients with GSDIa, high childhood TG was associated with an increased risk of HCA, and earlier onset of HCA development, independent of sex-associated hypertriglyceridaemia, and *G6PC1* genotype.

**Lay summary:**

Glycogen storage disease type Ia (GSDIa) is a rare, inherited metabolic disease that can be complicated by liver tumours (hepatocellular adenomas), which in turn may cause bleeding or progress to liver cancer. Risk factors associated with hepatocellular adenoma formation in patients with GSDIa are largely unknown. In our study, we found that high serum triglyceride concentrations during childhood, but not specific genetic variants, were associated with increased risk of hepatocellular adenoma diagnosis later in life.

## Highlights

• Glycogen storage disease Ia (GSDIa) is a metabolic disease caused by mutations in glucose-6-phosphatase catalytic subunit 1 (*G6PC1*).
• Patients with GSDIa often develop hepatocellular adenoma (HCA), with unclear risk factors.
• Metabolic control in GSDIa is commonly evaluated through serum triglyceride concentration (TG).
• Patients with GSDIa with high childhood TG had increased risk and earlier onset of HCA.
• Sex-associated hypertriglyceridaemia and *G6PC1* genotype were not associated with HCA.

## Graphical abstract

**Figure:**
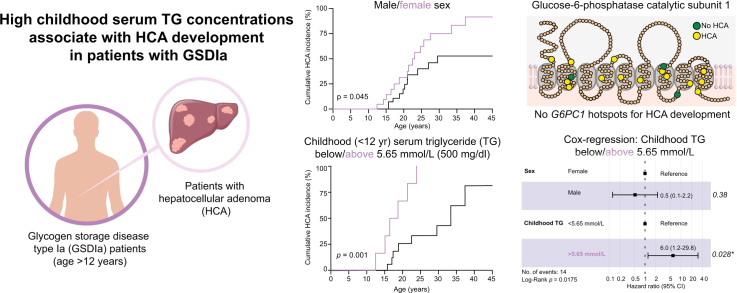

## Introduction

Glycogen storage disease type Ia (GSDIa; OMIM #232200) is a rare, inborn error of carbohydrate metabolism caused by pathogenic variants in the glucose-6-phosphatase catalytic subunit 1 (*G6PC1*) gene.^1^^,^^2^ The GSDIa phenotype is characterised clinically with fasting intolerance, hepatomegaly, and failure to thrive and biochemically with non-ketotic hypoglycaemia, and hypertriglyceridaemia. Evolving dietary strategies have greatly improved the life expectancy of patients with GSDIa, shifting the GSDIa paradigm from an acute and lethal disease to a chronic disorder. Long-term complications include hepatocellular adenoma (HCA) formation.[3], [4], [5]

HCAs are rare, benign liver tumours, with size (>5 cm)-dependent associated complications consisting of hepatic haemorrhage, or transformation to hepatocellular carcinoma.[6], [7], [8], [9] Outside the context of GSDIa, HCA formation is strongly associated with female sex, as >90% of HCAs occur in females, and with circulating oestrogen or androgen (*e.g.* oral contraceptives or anabolic steroids).[9], [10], [11] In GSDIa, however, about 30% of patients with HCA are male. HCA incidence in GSDIa increases with age, with a median age of diagnosis at around 15 years and an incidence of 70–80% over the age of 25 years.^3^^,^[12], [13], [14], [15]

*G6PC1* is a single-copy gene, with 5 exons coding for 357 amino acids.^2^ *G6PC1* expression is restricted to the liver, kidney, and intestine.^2^ Genetic variants within the *G6PC1* catalytic domain (amino acids 83, 119, 170, and 176) have shown to completely abolish glucose-6-phosphatase (G6Pase) function, whereas truncating (nonsense) variants either abolish or greatly impair G6Pase function.^2^ G6Pase dysfunction impairs hydrolysis of glucose-6-phosphate to glucose and phosphate, which disrupts the final and common step of glycogenolysis and gluconeogenesis.^5^^,^^16^ Although the *G6PC1* genotype has been linked to the severity of the metabolic phenotype of GSDIa, no specific *G6PC1* variants have definitively been associated with HCA formation.

Improved dietary management in GSDIa has resulted in improved metabolic control, which is commonly evaluated through serum triglyceride concentration (TG). Prolonged suboptimal metabolic control (hypertriglyceridaemia >5.65 mmol/L or 500 mg/dl) has been associated with HCA development.[5], [16] Recent studies on patients with GSDIa demonstrate better clinical outcomes, including lower TG and lower HCA prevalence compared with historical cohorts, which at least in part may be attributed to optimised dietary treatment strategies.^12^

Because longitudinal data on HCA incidence in GSDIa patients are scarce, the association and potential interaction of sex, *G6PC1* genotype, and metabolic control on HCA development is as yet unknown. The aim of this study was to assess the association between sex, type of *G6PC1* variants, TG in childhood, and HCA formation in a nationwide cohort of patients with genetically confirmed GSDIa.

## Patients and methods

### Study design

A nationwide, retrospective, observational, multicentre cohort study of patients with GSDIa was performed between 1969 and September 2021. The metabolic expert centres of 7 Dutch university medical centres provided information on patients followed-up. Inclusion criteria were as follows: current age ≥12 years, availability of diagnostic imaging, and GSDIa diagnosis based on *G6PC1* genetic analysis by traditional Sanger sequencing or next-generation sequencing. Strengthening the Reporting of Observational Studies in Epidemiology guidelines were adhered to for study design and manuscript preparation.^17^ The study protocol conformed to the ethical guidelines of the 1975 Declaration of Helsinki. The Law on Medical Scientific Research involving human beings (WMO) did not apply in an *a priori* approval by the Medical Ethical Committee of the University Medical Center Groningen (UMCG-MEC 2019-119). The study was registered before initiation in the UMCG research registry (UMCG-RR #202000465). All patient data were collected and processed in accordance with Dutch privacy laws.

### Data collection and definitions

HCA was diagnosed by either magnetic resonance imaging (MRI), histopathology, or both. The date of the first HCA diagnosis was retrospectively adjusted to the first tumour observation on ultrasound, in case of later diagnosis on MRI or histopathology. The largest HCA diameter was measured by ultrasound and/or MRI according to Response Evaluation Criteria in Solid Tumors version 1.1 (RECISTv1.1) criteria.^18^

*G6PC1* variants were categorised according to both the molecular characteristics of the genetic variants and the G6Pase location. All *G6PC1* missense variants in the active site (*i.e.* amino acids 83, 119, 170, and 176) and all *G6PC1* nonsense variants (regardless of location) were categorised as predicted severe variants (PSVs). Patients’ *G6PC1* genotypes were categorised as 0, 1, or 2 PSVs. *G6PC1* variants accounting for 50% or more of observed variants in the cohort (*i.e.* p.Arg83Cys, p.Gln347X, and p.Gln27ArgfsX9) were grouped and compared with all other variants.

Birth cohorts were defined as the older or current treatment era as previously reported.^12^ The current treatment was defined as treatment that started in 1986, the year when large-scale clinical use of uncooked cornstarch therapy commenced.^12^

TGs were measured at the local laboratories according to standard practice. TG data were expressed in mmol/L. Longitudinal childhood TGs were calculated as the median of measurements per 6 months per patient. Of included patients, a single childhood TG was calculated per patient as the median of all measurements obtained and available before the age of 12 years. Childhood TGs were categorised into low and high childhood TGs, defined as those patients with median childhood TG above or below 5.65 mmol/L (500 mg/dl), according to the previous definition.^15^ To correct for patients with metabolic dysregulation, and thereby with more frequent TG measurements, sensitivity analyses on childhood TG were performed by prior calculation of the median TG per 6 months and then calculating a single median TG on those values. Sensitivity analyses on childhood TG stratification were also performed through categorisation of childhood TG above or below 6.0 mmol/L, as recommended by the European Study on GSDIa management guideline.^3^^,^^4^ A sensitivity analysis on childhood TG and development of HCA was performed for use of lipid-lowering drugs (including fibrates, statins, omega-3 fatty acid supplements, or ursodeoxycholic acid) at any given time before HCA diagnosis.

### Data presentation and statistical analysis

Patients were categorised in groups according to sex (male/female), number of PSVs (0, 1, or 2, and 0/1 or 2), childhood TG (low/high), and birth cohort (older/current). Study data were collected from individual patient records and managed using REDCap electronic data capture tools (Vanderbilt University, Nashville, TN, USA) hosted at the UMCG.^19^^,^^20^ Genetic variants were presented according to Human Genome Variation Society recommendations.^21^ Figures were composed using R (R Foundation for Statical Computing, Vienna, Austria) and GraphPad Prism version 9.0 for Mac (GraphPad Software, La Jolla, CA, USA; www.graphpad.com). Dichotomous data were presented as proportions. Continuous variables were reported as median with IQR. Categorical variables were expressed as number (n) and percentage (%). Statistical analysis was performed using R version 4.1.0, including the ‘survival’ and ‘survminer’ packages. Univariate survival analyses were performed using Kaplan–Meier analyses and the log-rank test. Multivariate survival analyses were performed using Cox proportional hazards models. Parameters with 2-tailed *p* <0.05 were considered statistically significant.

## Results

**Fig. 1:**
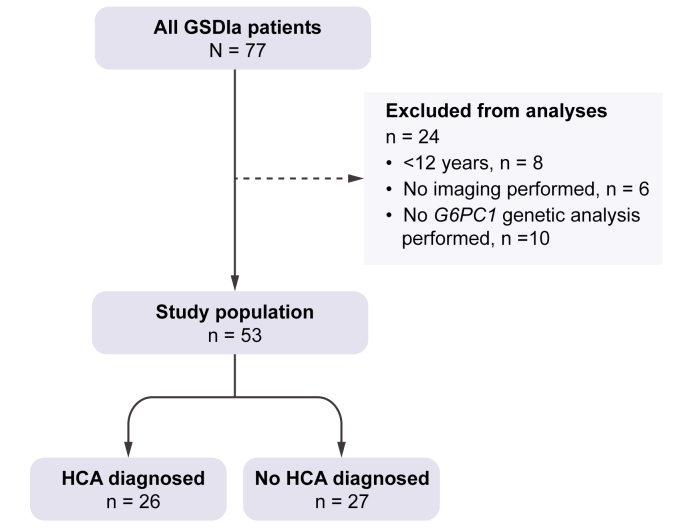
Flowchart of inclusion of the study population. *G6PC1*, glucose-6-phosphatase catalytic subunit 1; GSDIa; glycogen storage disease type Ia; HCA, hepatocellular adenoma.

Seventy-seven patients with GSDIa from 66 families were diagnosed at the outpatient clinic of the 7 participating centres. Twenty-four patients were excluded because of current age <12 years (n = 8), no imaging performed (n = 6), or no *G6PC1* variant analysis available (n = 10)*.* Fifty-three patients from 46 families were included for data analysis, with a median follow-up time of 32 (22–43) years (Fig. 1). Most patients (56%) were diagnosed within their first year of life, and the median age of GSDIa diagnosis was 10 (5–30) months (Table 1).

**Table 1:** **Baseline characteristics of patients with GSDIa**.

Characteristic	Total cohort (n = 53)	Patients with HCA (n = 26)	Patients without HCA (n = 27)	*p* value
Current age (years)	34 (24–45)	37 (28–45)	25 (22-42)	0.07
Sex, n of female patients (%)	23 (43)	15 (58)	8 (30)	0.039∗
Age of GSDIa diagnosis (months)	10 (5.0–30)	11 (4–48)	11 (5.0–23)	0.98
Birth cohort				
Born before 1986, n (%)	21 (40)	12 (46)	9 (33)	0.34
Born after 1986, n (%)	32 (60)	14 (54)	18 (67)	
Childhood TG (mmol/L)^a^	3.95 (3.18–5.79)	4.60 (4.03–7.84)	3.16 (2.33–3.37)	<0.001∗
Type of *G6PC1* variant^b^				
No PSV, n (%)	7 (13)	3 (12)	4 (15)	0.88
1 PSV, n (%)	11 (21)	6 (23)	5 (19)	
2 PSVs, n (%)	35 (66)	17 (65)	18 (67)	

Continuous values are provided as median and IQR.

*G6PC1*, glucose-6-phosphatase catalytic subunit 1; GSDIa, glycogen storage disease type Ia; HCA, hepatocellular adenoma; PSV, predicted severe variant; TG, serum triglyceride concentration.

a Median of TG up to and including 12 years of age.

b PSVs are any nonsense *G6PC1* variants and all missense variants within the *G6PC1* active site.

∗ Levels of significance: *p* <0.05 (Mann–Whitney *U* test and Chi-square test).

### GSDIa and HCA formation

**Fig. 2:**
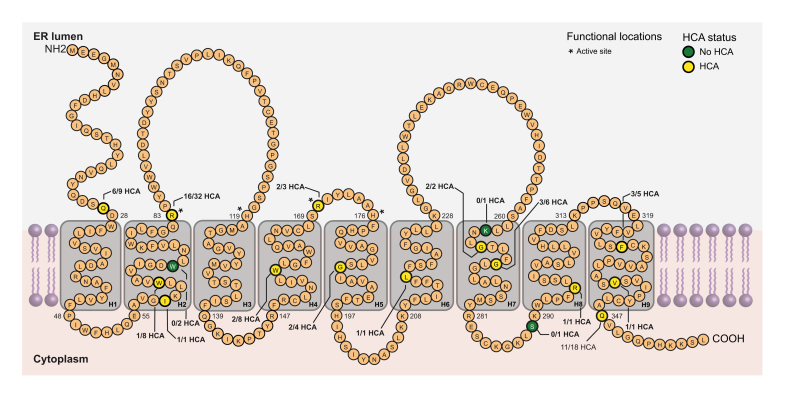
Schematic overview of *G6PC1* with number of observed variants and occurrence of HCA at each site. No specific *G6PC1* variant was associated with HCA formation. The *G6PC1* active site is defined as amino acids 83, 119, 170, and 176. *G6PC1*, glucose-6-phosphatase catalytic subunit 1; HCA, hepatocellular adenoma; ER lumen, endoplasmatic reticulum lumen.

HCA was diagnosed in 26 of 53 GSDIa patients (49%), at a median age of 21 (17–25) years. The lowest age of HCA diagnosis was 13 years (Fig. 2A). No difference was observed in age of GSDIa diagnosis between patients who did and those who did not develop HCA (*p* = 0.98; Table 1). Kaplan–Meier survival analysis demonstrated no significant difference in time to HCA development between birth cohorts before and after the introduction of uncooked corn starch diet in 1986 (Fig. 2B). Eight patients were diagnosed with hepatic adenomatosis (diagnosis of >10 HCAs). The median number of HCAs in patients without adenomatosis was 3 (1–6). The median diameter of all HCAs was 35 (18–65) mm (Supplementary Materials and methods; Table S1).

### HCA formation and sex

HCA formation was more common in female than in male patients with GSDIa (at age 25 years, 48% and 30%, respectively, log-rank *p* = 0.045; Fig. 2C and Table 1). HCA formation also occurred earlier in female patients; the age at which 50% of the women had developed HCA was 23 years, compared with 30 years in males (Fig. 2C). Adenomatosis was diagnosed in 3/13 male patients and 5/10 female patients (*p* = 0.69). Among patients without adenomatosis, male patients had a median of 6 (2–7) HCAs, compared with 3 (1–5) for females (*p* = 0.22). The largest median HCA size was 41 (15–104) mm in males and 28 (19–47) mm in females (*p* = 0.64).

### HCA formation and *G6PC1* gene variants

**Fig. 3:**
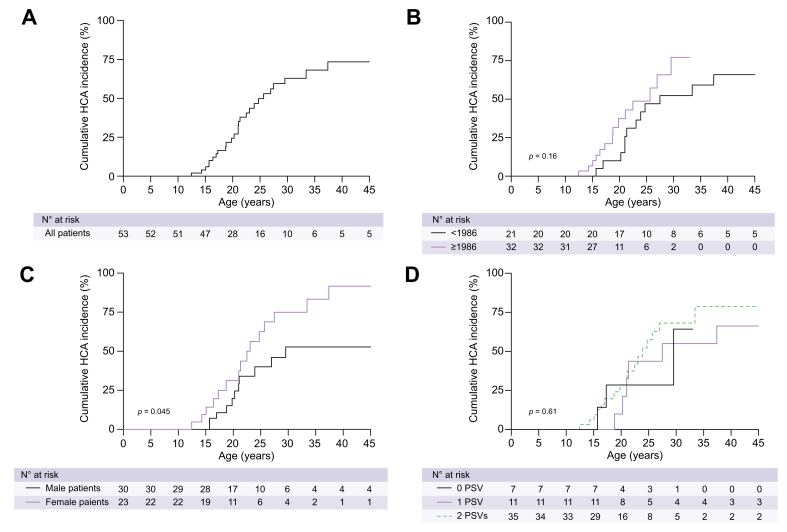
Kaplan–Meier survival analysis for time to HCA occurrence in patients with GSDIa. (A) Total cohort. (B) Stratified by treatment era. (C) Stratified by sex. (D) Stratified by *G6PC1* variant severity. Levels of significance: *p* values noted (log-rank test). *G6PC1*; glucose-6-phosphatase catalytic subunit 1; HCA, hepatocellular adenoma; GSDIa, glycogen storage disease type Ia; PSV, predicted severe variant.

Two-thirds of GSDIa patients carried 2 PSVs, 20% had 1 PSV, and 13% had no PSV (Supplementary Materials and methods; Tables S1 and S2). The number of PSVs within a patient with GSDIa was not associated with the diagnosis of HCA (*p* = 0.88; Table 1). The most frequently observed *G6PC1* variants were p.Arg83Cys (26%), p.Gln347X (17%), and p.Gln27ArgfsX9 (9%; Table 2). No specific *G6PC1* variant hotspot was associated with HCA formation (Fig. 3). In total, 23 out of 53 (44%) patients with GSDIa carried homozygous *G6PC1* variants. Twenty-seven unique genetic variant combinations were observed (Table 2).

**Table 2:** **Frequency of *G6PC1* variants in relation to sex and HCA formation in GSDIa patients**.

Genetic variant	*G6PC1* variant	Type of variant^a^	Frequency, n (%)	Female sex n (%)	HCA formation, n (%)
c.247C>T c.326C>T	p.Arg83Cys	PSV	28 (26)	14 (50)	14 (50)
c.1039C>T	p.Gln347X	PSV	18 (17)	11 (61)	11 (61)
c.79delC	p.Gln27ArgfsX9	PSV	9 (8.5)	4 (44)	6 (67)
c.189G>A	p.Trp63X	PSV	8 (7.6)	4 (50)	1 (13)
c.467G>T	p.Trp156Leu	Non-PSV	8 (7.6)	0 (–)	2 (25)
c.809G>T c.1039C>T	p.Gly270Val	Non-PSV	6 (5.7)	1 (17)	3 (50)
c.979_981delTTC c.980_982delTCT c.1058delTTC	p.Phe327del	PSV	5 (4.7)	2 (40)	3 (60)
c.248G>A	p.Arg83His	PSV	4 (3.8)	(50)	2 (50)
c.563G>C	p.Gly188Arg	Non-PSV	4 (3.8)	1 (25)	2 (09)
c.508C>T	p.Arg170X	PSV	3 (2.8)	2 (67)	2 (67)
c.209G>A	p.Trp70X	PSV	2 (1.9)	1 (50)	0 (–)
c.797G>T	p.Gly266Val	Non-PSV	2 (1.9)	1 (50)	2 (100)
c.IVS4+1G>A (c.562+10G>A, intron)	Unknown	PSV	2 (1.9)	1 (50)	0 (–)
2bp deletion exon 1	p.Ile59X	PSV	1 (0.9)	1 (100)	1 (100)
c.648G>T	p.Leu216Leu	PSV	1 (0.9)	1 (100)	1 (100)
c.788delA	p.Lys263ArgfsX38	PSV	1 (0.9)	0 (–)	0 (–)
c.866G>A	p.Ser289Asn	Non-PSV	1 (0.9)	0 (–)	0 (–)
c.884G>A	p.Arg295His	Non-PSV	1 (0.9)	1 (100)	1 (100)
c.1091G>T	p.Val338Phe	Non-PSV	1 (0.9)	1 (100)	1 (100)
Unknown	p.Arg380His	Non-PSV	1 (0.9)	0 (–)	0 (–)

*G6PC1*, glucose-6-phosphatase catalytic subunit 1; GSDIa, glycogen storage disease type Ia; HCA, hepatocellular adenoma; PSV, predicted severe variant.

a PSVs are any nonsense *G6PC1* variants and all missense variants within the *G6PC1* active site.

The number of PSVs was not associated with time to HCA formation (Fig. 2D), neither when comparing 0 PSV or 1 PSV, to 2 PSVs (Supplementary Materials and methods; Fig. S1A). Analyses of the three most frequently observed variants (p.Arg83Cys, p.Gln347X, and p.Gln27ArgfsX9) did not reveal any significant association with HCA occurrence, for mono-allelic, bi-allelic, and homozygous variants compared with other genetic variants in the cohort (Supplementary Materials and Methods; Fig. S1B–D).

### HCA formation and childhood TG

**Fig. 4:**
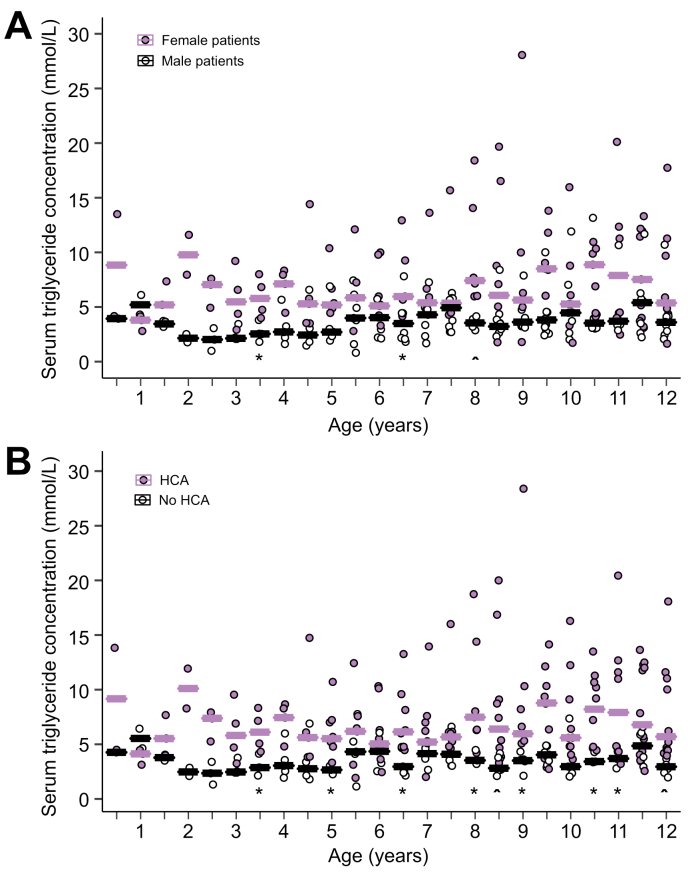
Longitudinal median childhood TG in patients with GSDIa per patient per 6 months. (A) Stratified for sex. (B) Stratified for diagnosis of HCA. Horizontal lines represent median TGs per moment of measurement. Levels of significance: ∗*p* <0.05; ^*p* <0.01 (Mann–Whitney *U* test). GSDIa, glycogen storage disease type Ia; HCA, hepatocellular adenoma; TG, serum triglyceride concentration.

Childhood TG data were available in 23 patients with GSDIa (10 females, 43%), with a median childhood TG of 3.9 (3.2–5.8) mmol/L. In total, 14 of the 23 patients developed HCA during follow-up. Male patients with GSDIa had a significantly lower median childhood TG than female patients with GSDIa (3.4 [3.0–4.2] *vs*. 5.6 (4.0–7.9) mmol/L, respectively; *p* = 0.026; Fig. 4A). The median childhood TG was 3.9 (3.3–4.2) mmol/L for patients with GSDIa with 0 PSV, 3.7 (3.4–3.9) mmol/L for those with 1 PSV, and 4.4 (3.2–7.8) mmol/L for those with 2 PSVs (0 *vs*. 1 PSV *p =* 0.79; 1 *vs*. 2 PSVs *p* = 0.65; 0 *vs*. 2 PSVs *p* = 0.38). Patients with GSDIa who developed HCA had a median childhood TG of 4.6 (4.0–7.8) mmol/L, compared with 3.2 (2.3–3.4) mmol/L for patients with GSDIa and without HCA diagnosis (*p* <0.001; Fig. 4B). Seventeen patients with GSDIa (74%) had a median childhood TG of <5.65 mmol/L (500 mg/dl).^15^ A sensitivity analysis with stratification of patients according to an alternative cutoff value of 6.0 mmol/L (proposed by Rake *et al.*^4^) yielded exactly the same patient distribution and similar outcome. In a separate sensitivity analysis on the historical use of lipid-lowering drugs or not, the median childhood TG was 3.7 (3.0–4.3) *vs*. 3.9 (3.2–7.3) mmol/L for patients with or those without history of lipid-lowering drug use, respectively (*p* = 0.56). Kaplan–Meier survival analysis did not reveal a significant difference in time to HCA diagnosis between patients with and those without historical use of lipid-lowering drugs (log-rank *p* = 0.18).

**Fig. 5:**
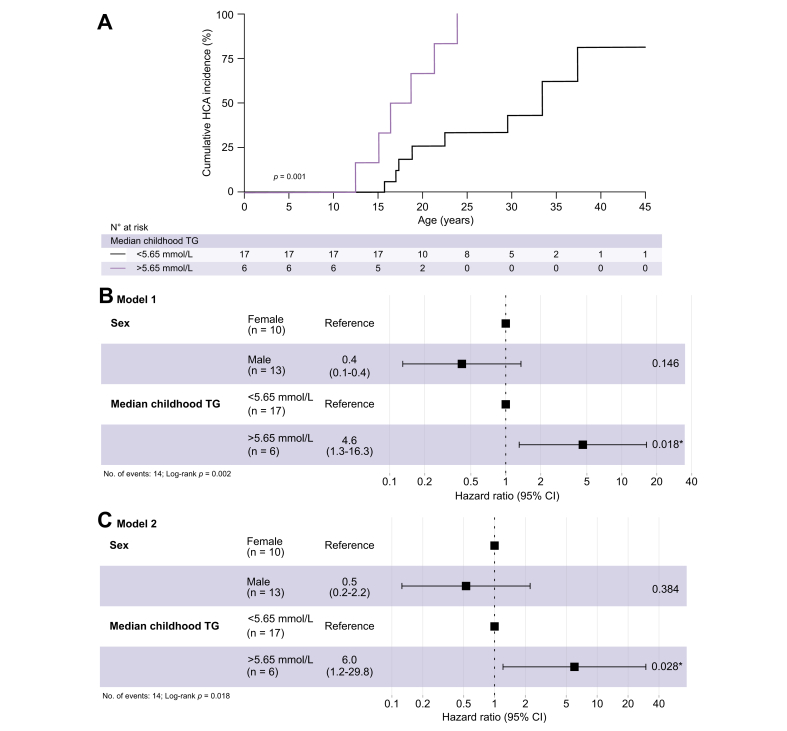
Influence of median childhood TG on HCA occurrence in patients with GSDIa, with patients clustered according to childhood TG above or below 5.65 mmol/L (500 mg/dl). (A) Kaplan–Meier survival analysis for time to HCA occurrence, stratified by median childhood TG above/below 5.65 mmol/L. Levels of significance: *p* values noted (log-rank test). (B) Cox regression analysis including sex and median childhood TG (model 1). Levels of significance: *p* values noted (Cox regression analysis). (C) Cox regression analysis including sex, median childhood TG, and interaction term for sex and median childhood TG (model 2). Levels of significance: *p* values noted (Cox regression analysis). GSDIa, glycogen storage disease type Ia; HCA, hepatocellular adenoma; TG, serum triglyceride concentration.

Fifty percent cumulative HCA incidence was 18 years for patients with GSDIa with a median childhood TG of >5.65 mmol/L, compared with 33 years for patients with GSDIa with a median childhood TG of <5.65 mmol/L (log-rank *p* = 0.001; Fig. 5A). A multivariate Cox regression model was performed, after testing the proportional hazard assumption using Schoenfield residuals. A model 1 including sex and categorised median childhood TG (above/below 5.65 mmol/L) was constructed (Fig. 5B). Male sex was associated with HCA formation with a hazard ratio (HR) of 0.4 (95% CI 0.1–1.4; *p* = 0.15). In this model, patients with GSDIa with a median childhood TG of >5.65 mmol/L had an HR of 4.6 (95% CI 1.3–16.3) for lifetime HCA development (*p* = 0.018). Because females had a higher median childhood TG than males (Fig. 4A), an interaction term was included in model 2 (Fig. 5C). In model 2, patients with GSDIa with a median childhood TG of >5.65 mmol/L had an HR of 6.0 (95% CI 1.2–29.8) for formation of HCA (*p* = 0.028).

## Discussion

We investigated potential risk factors for the development of HCA in patients with GSDIa, using a retrospective, nationwide, observational cohort. During a median follow-up time of 32 years, HCA developed in 26/53 patients. High childhood TG was observed more frequently in female GSDIa patients and was an independent risk factor for HCA development. We did not identify a clear *G6PC1* genotype association with HCA development.

Previous studies have shown that HCA formation in GSDIa occurs during adolescence, which is consistent with our current results.^3^^,^^15^ In our cohort of patients with GSDIa, by the age of 40 years, 65% of female patients and 37% of male patients had developed HCA, which is similar to previous reports.^9^^,^^14^^,^^22^ The higher frequency of HCA in male patients with GSDIa than that of HCA in male patients without GSDIa suggests an alternative, additional pathway to HCA genesis in addition to the exposure to high circulating oestrogen/androgen concentration caused by either increased endogenous production (in overweight patients) or supplementation (oral contraceptives or anabolic steroids).[9], [11], [23], [24] The lower childhood TG observed in male patients, compared with those in female patients, suggests that there may be an intricate relationship between sex-associated TG metabolism and HCA formation (Fig. 4a).

In our study, as well as in previous reports, HCA formation in GSDIa has especially been reported in patients with metabolic dysregulation (by either severe G6Pase dysfunction or therapy incompliance), whereas HCA regression has been observed after strict dietary management.^12^^,^^25^ It has been suggested that a Warburg-like metabolic switch in hepatocytes resulting from metabolic imbalance contributes to tumour development in GSDIa. The consequent hyperactivation of specific pathways inducing cell growth and mitotic activity may promote hepatic tumourigenesis in patients with GSDIa.^26^ For instance, enhanced fatty acid synthase activity in GSDIa may provide a beneficial environment for neoplastic progression, as many malignant tumours, including hepatocellular malignancies, display increased fatty acid synthase activity, whereas fatty acid synthesis inhibition has antitumoural effects.[27], [28], [29] However, whether cellular adaptations in metabolic and/or signal transduction pathways explain the increased risk for (advanced) HCA development in patients with severe G6Pase dysfunction or therapy incompliance remains to be established in future mechanistic studies.

There is only limited research on genotype–phenotype correlations for GSDIa.[1], [2], [3]^,^^15^^,^^30^ A thorough investigation of *G6PC1* genotype in relation to HCA development, however, has not been reported thus far. In our study, *G6PC1* variants and G6Pase impairment, indirectly quantified through the number of PSVs, were not significantly associated with HCA development. We also did not identify a ‘hotspot’ for pathogenic *G6PC1* variants that was associated with HCA development. No novel genetic variations were identified in this cohort, and more than half of patients were diagnosed with 2 PSVs.[31], [32], [33] PSV load, analysed individually (0 *vs.* 1 *vs.* 2 PSVs) or grouped (0/1 *vs.* 2 PSVs) did not reveal as a particular risk factor for HCA development.

Our cohort consists of 27 unique *G6PC1* variant combinations including 23 subjects with homozygous *G6PC1* variants. Although many patients in our study display unique combinations of *G6PC1* variants and despite a relatively low number of inclusions, lessons can be learned from patients with homozygosity for specific *G6PC1* variants. For example, the median age of HCA diagnosis was 16 (15–17) years for p.Arg83Cys homozygotes (n = 10), as compared with 27 (26–27) years for p.Gln347X homozygotes (n = 3; Supplementary Materials and methods; Tables S1 and S2). Patients with GSDIa exhibiting attenuated hypoglycaemic phenotypes may explain clinical GSDIa diagnosis at adult ages. We previously reported milder fasting intolerance in patients with GSDIa homozygous for c.467G>T (p.Trp156Leu) and c.1039C>T (p.Gln347X), *G6PC1* variants that are associated with retained G6Pase activity *in vitro*.^34^ By contrast, patients with GSDIa with compound heterozygosity, for c.508C>T (p.Arg170X) and c.575C>T (p.Ala192Val), homozygosity for c.1039C>T (p.Gln347X), and compound heterozygosity for c.648G>T (p.Leu216Leu) and c.986A>T (p.Lys329Met), resulting in reduced G6Pase activity *in vitro*, have presented clinically with hepatocellular carcinoma, HCA, or acute pancreatitis, respectively.[35], [36], [37] Similarly, patients homozygous for the common Japanese c.648G>T (p.Leu216Leu) *G6PC1* pathogenic splice variant are at increased risk of hepatocellular carcinoma.^30^^,^^38^^,^^39^ In summary, we hypothesise that the complex human GSDIa phenotype including HCA susceptibility is at least partially explained by the impact of the *G6PC1* genotype and the duration of the untreated, highly perturbed metabolic state, with subsequent late diagnosis, start of dietary treatment, and compliance.^40^

Dietary management strategies are the cornerstone of GSDIa treatment. Continuous glucose infusion, continuous nocturnal drip, and uncooked cornstarch have greatly improved GSDIa outcomes.[3], [4], [12] TG is considered an important longitudinal outcome parameter for biomedical control in GSDIa. The 2002 European Study on GSDIa management guideline recommends TG <6.0 mmol/L as a biomedical target, after performing a large multicentre observational cohort study evaluating GSDIa clinical course and outcomes.[3], [4] The 2010 Association for Glycogen Storage Disease Conference consensus panel discussion defined a TG target at 500 mg/dl (5.65 mmol/L), and were used as a stratification by Wang *et al.*^15^ In our study, childhood TG levels >5.65 mmol/L were associated with increased risk of HCA development as well as earlier HCA diagnosis. Stratification of the cohort at 6.0 mmol/L, as defined by the European guidelines, yielded similar results.^4^ Our observations confirm the results from Wang *et al.*^15^ that high TG precedes HCA diagnosis in patients with GSDIa, which should re-emphasise the importance of strict metabolic management to prevent (or delay) HCA formation. In the aforementioned paper, a 5-year mean TG before HCA diagnosis or censoring was calculated. Our current results, however, show that HCA formation may already be predicted during childhood, although our dataset does not allow us to differentiate between metabolic control and controllability (because of genotype or sex) of patients. Our observation of comparable median childhood TGs between 0, 1, and 2 PSVs suggests mainly female sex as a non-therapeutic risk factor for hypertriglyceridaemia.

Several limitations may have influenced the outcomes of this study. First, (availability of) treatment strategies, including dietary therapy, have evolved over time. Increased treatment efficacy influenced both metabolic control and overall survival of patients, including those with more severe metabolic phenotypes. However, stratification by birth cohort did not reveal any significant differences in HCA occurrence. Second, patient-specific heterogeneity in environmental and/or genetic factors may have resulted in residual confounding that could not be accounted for owing to the retrospective nature of this study. Third, TG analysis is likely more often performed in patients with metabolic dysregulation, as this parameter is measured frequently during hospital admissions or outpatient department evaluations. We have mitigated this aspect by calculating medians for all childhood TG data, and a median per 6 months for longitudinal measurements, yielding similar results.

The data in this study may assist in patient-centred (dietary) management and follow-up, as we have identified subgroups of patients especially vulnerable for HCA development. This study illustrates the importance of correlating the multifactorial processes that define the complex human GSDIa phenotype, including the *G6PC1* genotype, parameters of biomedical control, and sex to long-term complications. We have analysed a subset of those traits, and more investigations are needed on the alternate complications such as nephropathy and biomedical outcome markers such as lactate, uric acid, and continuous glucose monitoring parameters. These are urgently warranted to compose a set of person-centred outcomes for patients with GSDIa, to standardise future data collections, to identify important endpoints for clinical trials, and to evaluate novel treatments in the future.[41], [42], [43]

In conclusion, in patients with GSDIa, high childhood TG was associated with an increased risk of HCA, and earlier onset of HCA development, independent of sex-associated hypertriglyceridaemia, and *G6PC1* genotype. Recognition of these risk factors may assist in further development of individual monitoring strategies for GSDIa.

## Financial support

FP was supported by a Junior Scientific Masterclass grant from the 10.13039/501100001721University of Groningen as a MD/PhD (MD-PhD 16-24). MHO was supported by a VIDI grant (917.15.350) from the 10.13039/501100003246Dutch Research Council and holds a Rosalind Franklin Fellowship from the 10.13039/501100001721University of Groningen.

## Authors’ contributions

Study design: MPDH, FP, MHO, HJV, TGJD, VEM. Data collection: MPDH, FP. Analysis: MPDH, FP. Drafting of manuscript: MPDH, FP, MHO, HJV, TGJD, VEM. Drafting of figures: MPDH, FP. Feedback on data analyses: MHO, HJV, TGJD, VEM. Feedback on the manuscript: MCGJB, CEMH, MCHJ, JGL, ML, AJMR, MAEMW. Assistance with data collection: MCGJB, CEMH, MCHJ, JGL, ML, AJMR, MAEMW.

## Data availability statement

The data that support the findings of this study are available from the corresponding author, VEM, upon reasonable request.

## Conflicts of interest

There are no conflicts of interest to be reported**.**

Please refer to the accompanying ICMJE disclosure forms for further details.
