# Supplementary material for: High childhood serum triglyceride concentrations associate with hepatocellular adenoma development in patients with glycogen storage disease type Ia
Source: JHEP Rep. 2022 May 29;4(8):100512. doi: 10.1016/j.jhepr.2022.100512 (PMC9263528; doi:10.1016/j.jhepr.2022.100512)
Supplement: Multimedia component 1 [file mmc1.pdf]

High childhood serum triglyceride concentrations associate with hepatocellular adenoma development in patients with glycogen storage disease type Ia

Martijn P.D. Haring, Fabian Peeks, Maaike H. Oosterveer, Martijn C.G.J. Brouwers, Carla E.M. Hollak, Mirian C.H. Janssen, Janneke G. Langendonk, Alexander J.M. Rennings, Margreet A.E.M. Wagenmakers, Henkjan J. Verkade, Terry G.J. Derks, Vincent E. de Meijer

Table of contents

Fig. S1..2

Table S1..3

Table S2..4

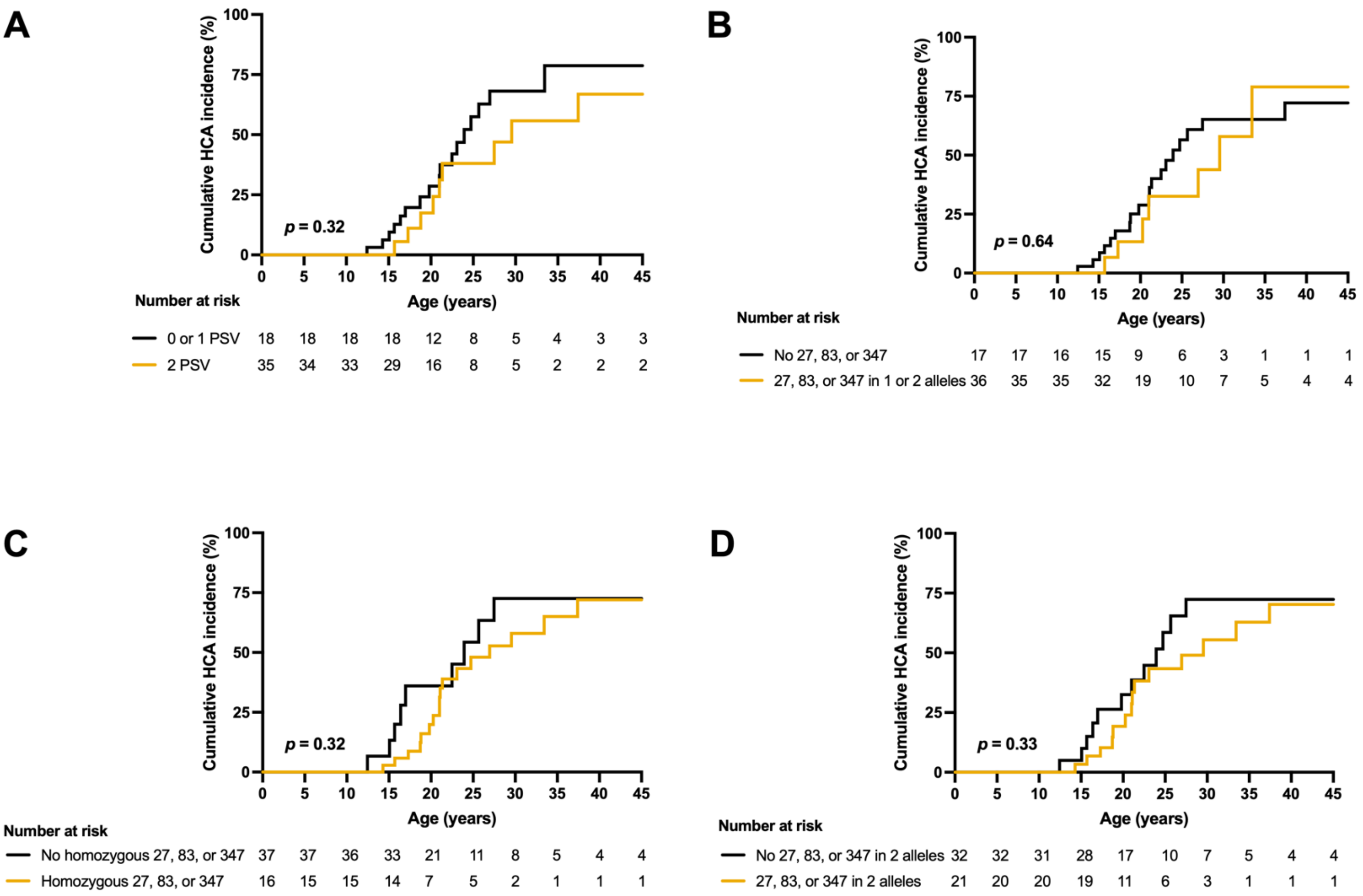

Fig. S1. Influence of most frequently observed *G6PC1* variants on HCA occurrence in GSDIa patients. (A) Stratified for 0 & 1 vs. 2 PSV. (B) Stratified for at least 1 variant in *G6PC1* amino acid number 27, 83, or 347. (C) Stratified for homozygous *G6PC1* variant in amino acid number 27, 83, or 347. (D) Stratified for 2 *G6PC1* variants in amino acid number 27, 83, or 347 (not homozygous). Abbreviations: *G6PC1*, glucose-6-phosphatase catalytic subunit 1; HCA, hepatocellular adenoma; GSDIa, glycogen storage disease type Ia; PSV, predicted severe variant.

Table S1. *G6PC1* Genotype and phenotype of GSDIa patients with HCA

ID	Sex	*G6PC1* variant (allele 1 & 2)*	Familial geographical background	Current age (years)	Age of GSDIa diagnosis (months)	Age HCA diagnosis (years)	Diagnostic modality	Number of HCA†	Largest HCA size (mm)	Median childhood TG (mmol/L)‡
1	F	**p.Arg83His**<br>**p.Gln347X**	German	52	11	23	PA	>10	41	Not available
2	F	**p.Gln347X**<br>**p.Ilee59X**	Dutch	27	10	19	PA	1	17	9.6
3	F	**p.Gln27ArgfsX9**<br>**p.Gln27ArgfsX9**	Dutch	27	5	16	PA	5	45	8.0
4	F	**p.Trp63Ter**<br>p.Val338Phe	Dutch	52	175	21	PA	3	49	Not available
5	M	p.Gly266Val<br>**p.Phe327del**	Dutch	42	1	20	MRI	6	109	Not available
6	F	**p.Arg83Cys**<br>**p.Gln347X**	Dutch	54	13	25	PA	7	180	Not available
7	M	**p.Arg83Cys**<br>**p.Arg83Cys**	Moroccan	45	8	17	PA	>10	110	3.0
8	F	**p.Arg83Cys**<br>**p.Arg83Cys**	Turkish	28	4	15	PA	>10	60	6.8
9	F	**p.Arg83Cys**<br>**p.Arg83Cys**	Turkish	36	0	22	PA	1	43	4.4
10	M	p.Trp156Leu<br>p.Trp156Leu	Dutch	40	93	30	MRI	6	30	4.3
11	F	**p.Gln27ArgfsX9**<br>p.Gly266Val	Dutch	37	5	21	MRI	6	15	13.3
12	M	p.Gly188Arg<br>**p.Gln347X**	German	45	117	19	MRI	9	100	3.9
13	M	**p.Gln27ArgfsX9**<br>**p.Arg83Cys**	Hungarian	29	25	21	MRI	1	120	Not available
14	F	**p.Arg170X**<br>**p.Phe327del**	Dutch	37	3	33	MRI	1	10	4.3
15	M	**p.Arg83Cys**<br>**p.Arg83Cys**	Italian	45	3	16	US	>10	70	Not available
16	F	**p.Arg83Cys**<br>**p.Arg83Cys**	Greek	34	5	12	MRI	>10	21	7.9
17	F	p.Gly270Val<br>**p.Gln347X**	Dutch	45	1	37	US	2	19	3.7
18	M	**p.Arg170X**<br>**p.Phe327del**	Dutch	34	25	27	US	1	10	Not available
19	F	**p.Gln347X**<br>**p.Gln347X**	Moroccan	31	309	26	MRI	>10	48	Not available
20	M	p.Gly270Val<br>p.Gly270Val	Turkish	17	2	16	MRI	>10	41	4.8
21	F	p.Arg295His<br>p.Gly188Arg	Dutch	25	216	17	MRI	>10	28	3.9
22	F	**p.Arg83His**<br>**p.Leu216Leu**	Chinese	19	48	14	MRI	3	18	Not available
23	M	**p.Arg83Cys**<br>**p.Gln347X**	German	24	Not available	20	MRI	4	6	Not available
24	F	**p.Gln347X**<br>**p.Gln347X**	Moroccan	52	168	27	MRI	3	28	Not available
25	M	**p.Gln27ArgfsX9**<br>**p.Gln27ArgfsX9**	Dutch	44	5	24	MRI	6	104	7.7
26	M	**p.Arg83Cys**<br>**p.Gln347X**	Dutch	41	30	21	MRI	>10	21	Not available

*Bold genetic variants are predicted severe variants. †Adenomatosis is defined as diagnosis of >10 HCA. ‡Median of serum triglyceride concentration up to 12 years old.
Abbreviations: *G6PC1*, glucose-6-phosphatase catalytic subunit 1; HCA, hepatocellular adenoma; GSDIa, glycogen storage disease type Ia; US, ultrasound; MRI, magnetic resonance imaging; PA, histopathology; TG, triglyceride concentration.

Table S2. ***G6PC1*** **Genotype and phenotype of GSDIa patients without HCA.**

ID	Sex	*G6PC1* variant[*]	Familial geographical background	Current age (years)	Age of GSDIa diagnosis (months)	Median childhood TG (mmol/L)[†]
27	F	**p.Arg170X**<br>**p.Phe327del**	Dutch	52	13	Not available
28	M	**p.Gln27ArgfsX9**<br>**p.Lys263ArgfsX38**	Dutch	25	225	Not available
29	F	**p.Arg83Cys**<br>**p.Gln347X**	German	51	8	Not available
30[a]	M	p.Gly270Val<br>**p.Gln347X**	German	49	21	Not available
31[a]	M	p.Gly270Val<br>**p.Gln347X**	German	49	21	Not available
32	M	**p.Arg83His**<br>**p.Arg83His**	German	45	175	2.33
33	M	p.Gly270Val<br>**p.Gln347X**	Dutch	49	10	3.37
34	M	**p.Trp70X**<br>**p.Phe327del**	Dutch	23	25	Not available
35	M	**p.Trp63X**<br>**p.Trp63X**	Dutch	23	8	Not available
36[b]	M	p.Trp156Leu<br>p.Trp156Leu	Dutch	35	31	3.16
37[b]	M	p.Trp156Leu<br>p.Trp156Leu	Dutch	25	5	2.87
38[b]	M	p.Trp156Leu<br>p.Trp156Leu	Dutch	29	5	3.77
39	M	**p.Gln347X**<br>**p.Arg83Cys**	Dutch	24	42	Not available
40	F	**p.Arg83Cys**<br>**p.Arg83Cys**	Turkish	26	10	Not available
41	M	p.Arg380His<br>p.Gly188Arg	German	38	175	Not available
42	M	**p.Arg83Cys**<br>**p.Arg83Cys**	German	52	Not available	Not available
43	M	**p.Arg83Cys**<br>p.Ser289Asn	German	19	11	2.28
44	F	**p.Gln347X**<br>**p.Gln347X**	Dutch	19	22	1.77
45	M	**p.Arg83Cys**<br>**p.Arg83Cys**	Italian	37	12	4.17
46	F	**p.Arg83Cys**<br>**p.Arg83Cys**	Moroccan	37	0	Not available
47[c]	M	**p.Trp63X**<br>**c.562+10G>A (intron)**[‡]	Dutch	19	5	Not available
48[c]	F	**p.Trp63X**<br>**c.562+10G>A (intron)**[‡]	Dutch	14	0	Not available
49	M	**p.Arg83Cys**<br>p.Gly188Arg	German	23	Not available	Not available
50	F	**p.Gln27ArgfsX9**<br>**p.Trp70X**	Dutch	19	Not available	Not available
51	F	**p.Arg83Cys**<br>**p.Arg83Cys**	Turkish	21	1	Not available
52	M	**p.Gln27ArgfsX9**<br>**p.Trp63X**	Dutch	18	0	Not available
53	M	**p.Trp63X**<br>**p.Trp63X**	Dutch	23	8	Not available

[*]Bold genetic variants are predicted severe variants. [†]Median of serum triglyceride concentration up to 12 years old. [‡]Amino acid alteration not indexed. [a,b,c] denote siblings in different families.

Abbreviations: *G6PC1*, glucose-6-phosphatase catalytic subunit 1; GSDIa, glycogen storage disease type Ia; HCA, hepatocellular adenoma; TG, triglyceride concentration.
